# Disrupted Rich Club Organization of Hemispheric White Matter Networks in Bipolar Disorder

**DOI:** 10.3389/fninf.2020.00039

**Published:** 2020-08-26

**Authors:** Dandan Li, Weichen Liu, Ting Yan, Xiaohong Cui, Zehua Zhang, Jing Wei, Yunxiao Ma, Nan Zhang, Jie Xiang, Bin Wang

**Affiliations:** ^1^College of Information and Computer, Taiyuan University of Technology, Taiyuan, China; ^2^Translational Medicine Research Center, Shanxi Medical University, Taiyuan, China

**Keywords:** bipolar disorder, white matter connections, graph theory approach, hemispheric lateralization, rich club organization

## Abstract

Neuroimaging studies suggest disrupted connections of the brain white matter (WM) network in bipolar disorder (BD). A group of highly interconnected high-density structures, termed the ‘rich club,’ represents an important network for brain functioning. Recent works have revealed abnormal rich club organization in brain networks in BD. However, little is known regarding changes in the rich club organization of the hemispheric WM network in BD. Forty-nine BD patients and fifty-five age- and sex-matched normal controls (NCs) underwent diffusion tensor imaging (DTI). Graph theory approaches were applied to quantify group-specific rich club organization and nodal degree of hemispheric WM networks. We demonstrated that rich club organization of hemispheric WM networks in BD was disrupted, with disrupted feeder and local connections among hub and peripheral regions located in the default mode network (DMN) and the control execution network (CEN). In addition, BD patients showed abnormal asymmetry in the feeder and local connections, involving the hub and peripheral regions associated with emotion regulation and visuospatial functions. Moreover, the clinical symptoms of BD showed a significant correlation with the aberrant asymmetry in the regional degree of peripheral regions. These findings reveal that BD is closely associated with disrupted feeder and local connections but no alteration in rich-club connections in the rich club organization of hemispheric WM networks and provide novel insight into the changes of brain functions in BD.

## Introduction

Hemispheric lateralization refers to the asymmetry of the two brain hemispheres in terms of their anatomy and function (Toga and Thompson, 2003; Parker et al., 2005; Yasser et al., 2011). This feature is thought to have originated from evolutionary, developmental, experiential and pathological factors (Toga and Thompson, 2003) and is a prominent characteristic of human brain development. Abnormal hemispheric lateralization has long been proposed to be a consequence of altered neurodevelopment in individuals with psychotic disorders (Ho et al., 2017). Studies on white matter (WM) have shown that aberrant brain region asymmetries are highly correlated with disturbed functions such as executive function (Yin et al., 2013), emotion (Schwartz et al., 1975; Schulte et al., 2012), and language (O’Donoghue et al., 2017). Moreover, neuroimaging studies (Torgerson et al., 2013) have indicated that the WM structure in the brain is fundamental and crucial to brain function. Therefore, analyzing WM lateralization abnormalities might benefit the understanding of the underlying nature of brain function abnormalities in patients with psychiatric disorders and potentially help to elucidate disorder etiologies.

As one of the most distinct syndromes in psychiatry, bipolar disorder (BD) is mainly characterized as episodic elevations in emotion and disturbances in cognition function (Quraishi and Frangou, 2002; Saunders and Goodwin, 2010), affecting approximately 1% of the population (Belmaker, 2004; Collin et al., 2016). Accumulated structural studies (Bruno et al., 2008; Vivian et al., 2009; Mahon et al., 2013) have revealed abnormal asymmetries in the WM volume in BD patients. Moreover, lower fractional anisotropy (FA) in the right anterior cingulate gyrus [ACG] (Liu et al., 2010) and the right precuneus [PCUN] (Elisa et al., 2016) regions were observed in BD patients compared with normal controls (NCs). Notably, the ACG region is involved in cognitive and emotional processing (Bush et al., 2000), and the PCUN plays a role in regulating the memory function (Cavanna and Trimble, 2006). These studies may suggest that abnormal WM lateralization is a key factor in the manifestation of BD symptoms (Torgerson et al., 2013).

Complex network analysis combines diffusion tensor imaging (DTI) to model the brain as two hemispheric WM networks and examine the differences in WM organization between the left and right hemisphere networks (Yasser et al., 2011). Using graph theory approaches, accumulated evidence has revealed hemispheric asymmetries in the graph metrics of the brain WM network (Karen and Alexander, 2014; Zhong et al., 2016). Moreover, previous studies (Silk et al., 2016; Yang et al., 2017; Zhong et al., 2016; Li et al., 2018) have hypothesized that abnormal brain network asymmetry is linked to neuropsychiatric disorders. Accordingly, our prior study (Wang B. et al., 2018) using graph theory showed reduced hemispheric asymmetry in the topological organization of the brain WM networks of BD patients, such as global efficiency and small-worldness, suggesting the disrupted asymmetry of WM connections in BD.

The rich club organization, defined as a tendency for hub (high-degree) regions to be more densely connected among themselves than to peripheral (low-degree) regions (Power et al., 2013; Van den Heuvel and Sporns, 2013), is one of the key graph theory metrics that provides important information on the higher-level topology of brain networks (Van den Heuvel and Sporns, 2011; Collin et al., 2013). Recent studies on whole-brain WM networks (Collin et al., 2016; O’Donoghue et al., 2017; Wang Y. et al., 2018) have reported that the putamen [PUT], PCUN and insula [INS] are defined as hub regions and the rolandic operculum [ROL], inferior temporal gyrus [ITG], superior occipital gyrus [SOG], and lingual gyrus [LING] regions are defined as peripheral regions in BD patients. Notably, decreased asymmetry in the nodal efficiency of the ROL, ITG, SOG and LING has been revealed in BD patients (Wang B. et al., 2018). These findings may imply that abnormal hemispheric asymmetry in the rich club organization in BD occurs mainly through reduced WM connectivity in peripheral brain regions. However, it is unknown whether there are changes in the rich club organization of the hemispheric WM networks in BD patients.

This work used DTI data from 49 BD patients and 55 age- and sex-matched NCs to construct hemispheric WM networks. Graph theory approaches were used to analyze the network topology. Two connectivity measures (density and strength) and the nodal degree were used to assess the property of rich club organization. We aimed to investigate how the patterns of rich club organization change in hemispheric WM networks in BD patients. This study may serve a functional role in clinical trials and interventions for BD.

## Materials and Methods

### Subjects

All the subjects participating in the current study, including 49 BD patients and 55 age- and sex-matched NCs, were screened from the LA2K study. The detailed demographic and clinical characteristics for all the subjects are presented in Table 1. The Handscore described the handedness of subjects. It was obtained using a formula (Right + Left)/(Right - Left). Patient symptoms were evaluated using the 17-item Hamilton Depression Rating Scale (HAMD) (Hamilton, 1960) and the Young Mania Rating Scale (YMRS) (Young et al., 1978). The neuroimaging dataset was obtained via the publicly available OpenfMRI database^1^. This study was approved by Institutional Review Board of the University of California, Los Angeles (UCLA).

**TABLE 1:** Demographic and clinical characteristics of the samples.

Demographic characteristics	NC	BD	Statistics	
	*n* = 55	*n* = 49		
Age (years)	21–49 (33.7 ± 9.1)	22–50 (33.9 ± 9.0)	F = 0.209^a^	*p* = 0.649
Male/Female	27/28	28/21	F = 0.674^b^	*p* = 0.412
Duration of illness (months)	N/A	0–24 (2.1 ± 5.2)		
Medication dose (mg/day)	N/A	0–6210 (784.8 ± 1035.3)		
Handscore	0.80–1 (0.95 ± 0.09)	0.75–1 (0.93 ± 0.1)	F = 0.926^ a^	*p* = 0.338
YMRS	N/A	0–41 (11.9 ± 11.0)		
HAMD	N/A	0–32 (12.0 ± 8.4)		

*^a^Statistical comparison was performed using analysis of variance; ^b^Statistical comparison was performed using a chi-square test.*

### Data Preprocessing and Network Construction

Structural MRI data were obtained using 3T Siemens Trio scanners located at the Ahmanson-Lovelace Brain Mapping Center and the Staglin Center for Cognitive Neuroscience at UCLA. High-resolution 3D echoplanar imaging was acquired with the following parameters: repetition time (TR) = 1.9 s, echo time (TE) = 2.26 ms, flip angle = 90°, field of view (FOV) = 250 × 250 mm^2^, acquisition matrix = 256 × 256, sagittal plane, slice thickness = 1 mm, and 176 slices. Diffusion weighted imaging (DWI) data were collected using an echoplanar sequence with the following parameters: 64 directions, slice thickness = 2 mm, TR = 9 s, TE = 93 ms, 1 average, acquisition matrix = 96 × 96, flip angle = 90°, axial slices, and b = 1000 s/mm^2^.

Data preprocessing and network construction were performed using the MATLAB toolbox named pipeline for analyzing brain diffusion images (PANDA_1.3.1, http://www.nitrc.org/projects/panda). The data preprocessing procedure includes corrections for simple head movements and eddy current distortions. The FA of each voxel was computed, with higher values indicating more directionally restricted diffusion of water molecules. Briefly, individual T1-weighted images were coregistered to the b0 images in the DTI space. The transformed T1 images were segmented into WM and then non-linearly transformed to the International Consortium of Brain Mapping (ICBM) 152 T1 template in the Montreal Neurological Institute (MNI) space. The inverse transformations were used to warp the automated anatomical labeling (AAL) atlas from the MNI space to the DTI native space. Finally, for each individual DTI dataset, deterministic fiber tracking algorithms were used to reconstruct the WM pathways. In the brain mask, 7 seeds followed the main diffusion direction from voxel to voxel. The tractography was terminated when it reached a voxel with an FA value less than 0.1 or when the angle was greater than 35°. Based on the reconstructed fiber tracts, the WM connection between a pair of nodes was adopted if the fiber number (FN) was larger than 3 (Shu et al., 2011; Li et al., 2018; Wang et al., 2019). We selected the threshold value for the FN > 3 was to reduce pseudo-connections due to possible noise effects on the whole-brain WM tractography (Sun et al., 2015).

The whole-brain WM network was constructed for each subject based on AAL (Tzourio-Mazoyer et al., 2002) atlas. We normalized the AAL atlas to eliminate the hemispheric asymmetry effect of brain structure according to the methods proposed by Yan et al. (2009). Using the normalized AAL atlas, the whole-brain was divided into 90 regions (45 regions in each hemisphere). The node of structural network was defined as one region of the normalized AAL atlas. The weights of the structural network was defined the mean FA values of the connected fibers between 2 regions (Li et al., 2018; Yan et al., 2018; Wang et al., 2019). The reason for choosing FA is that it is an important indicator commonly used to examine the microstructure aspects of brain WM connections (Cui et al., 2013). Finally, a weighted 90 × 90 whole-brain anatomical network was constructed for each subject.

### Graph Theory Analysis of the Hemispheric Network

Based on the weighted 90 × 90 whole-brain WM network, we discarded inter-hemispheric WM connections and separated the whole-brain network into two 45 × 45 hemispheric networks for each subject. The graph metrics including the rich club organization and nodal degree were computed to evaluate the topological structure of all hemispheric WM networks. The nodal degree measures the number of edges connected to the node. This work used the GRETNA toolbox^2^ (Wang et al., 2015) in MATLAB to analyze the network topology. The results of the network analyses were visualized using the BrainNet Viewer toolbox^3^ (Xia et al., 2013).

#### Description and Measurement of Rich Club Organization

Rich club organization describes the central backbone for global communication in the brain network (Van den Heuvel and Sporns, 2011, 2013), which refers to nodes with higher degrees within brain networks (Van den Heuvel and Sporns, 2011). The rich club organization was described based on the normalized rich club coefficients (RCs). The normalized RC was greater than 1 over a range of degrees, indicating the existence of a rich club organization in the brain connectome. The RC is computed as the sum of the weights of the subset of connections larger than *k* in the network divided by the sum of the set of the strongest connections in the total network. The normalized RC was computed by normalizing the RC relative to a set of 1000 comparable random networks for each subject. In order to preserve the same number of row and column as a real weighted structural network, this paper used the Maslovs wiring algorithm (Maslov and Sneppen, 2002) to generate a random network. In addition, the strength and the degree distribution of structural network was preserved in the random network.

The current study selected hub regions based on the group-average FA network. The top 7 high-degree regions corresponding to the highest-ranking 16% of nodes were identified as hub nodes for each individual subject. The remaining 38 regions were identified as peripheral nodes. Once the nodes were classified into hub nodes and peripheral nodes, the edges of the network were classified as rich-club connections between two hub nodes, feeder connections from one hub node to one peripheral node, or local connections between two peripheral nodes (Van den Heuvel and Sporns, 2011; Van den Heuvel et al., 2012). Notably, the present study used two connectivity measures, the connectivity density and connectivity strength, to distinguish three types of connections in the rich club organization of hemispheric WM networks. The connectivity density describes the proportion of connections out of the total number of possible edges in the hemispheric WM network. The connectivity strength is calculated as the total sum of the weighted FA values of all the connections in the hemispheric WM network.

#### Hemispheric Asymmetry in the Graph Metrics

This paper applied the formula *AS*(*X*) = 100 × [*X*(*R*)-*X*(*L*)]/[*X*(*R*) + *X*(*L*)] (Yasser et al., 2011; Wang et al., 2019) to estimate the hemispheric asymmetry of the network topology graph metrics, where *X*(*R*) and *X*(*L*) refer to the graph metric for the right and left hemispheric networks, respectively. A negative *AS*(*X*) value indicates leftward asymmetry in the graph metric of *X*, while a positive *AS*(*X*) value demonstrates a rightward advantage in the graph metric of *X*.

### Statistical Analysis

All statistical analyses were performed using the Statistical Package for Social Science (SPSS, v19.0)^4^. For the group differences in the demographic characteristics, two-sample two-tailed *t*-tests were used for age and the handscore of subjects, and chi-square tests were used for sex. For both hemisphere and group differences in the graph metrics, repeated-measures analysis of variance (ANOVA) was employed with group as a between-subject factor and hemisphere (left and right) as the repeated-measures factor. Moreover, the hemisphere-by-group interaction was also considered. When the value of *p* < 0.05 (no correction for connectivity measures and Bonferroni-correction for nodal degree), the effect was statistically significant and *post hoc* tests (paired *t*-tests for hemisphere differences and independent *t*-tests for group differences) were performed. In this paper, the symbol ^∗^ indicates the value of *p* is smaller than 0.05. The symbol ^∗∗^ indicates the value of *p* is smaller than 0.01. The symbol ^∗∗∗^ indicates the value of *p* is smaller than 0.001.

This work also examined the correlation between the graph metrics and the symptom severity of BD patients by calculating Spearman correlation coefficient. Relationships were considered significant for uncorrected values of *p* < 0.05 because these correlations were exploratory in nature. For all statistical analyses, age, sex and handscore were treated as covariates.

## Results

### Rich Club Organization

**FIGURE 1:**
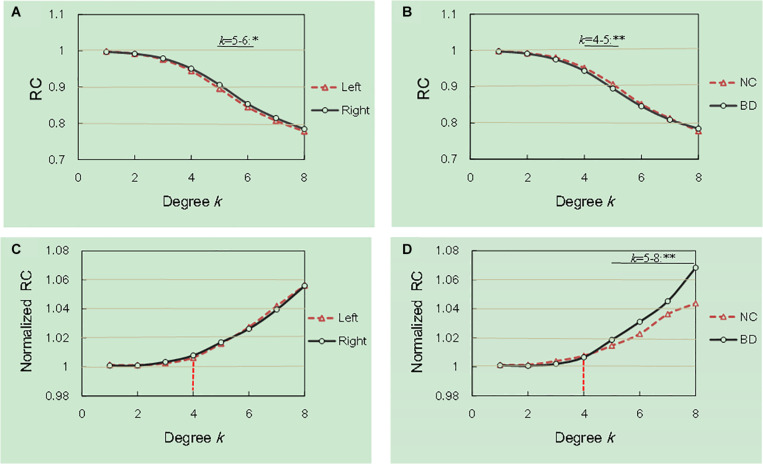
Hemisphere and group differences in both the RCs and normalized RCs. **(A,B)** depicted significant hemisphere and group differences in RC, respectively. **(C,D)** depicted significant hemisphere and group differences in normalized RC, respectively. *p < 0.05; **p < 0.01; ***p < 0.001.

We examined the hemisphere and group effects on the RCs and the normalized RCs, as shown in Figure 1. The Figure 1 described the mean degree of per group after running RC procedure per subject. Notably, we reported the degree levels only for the two groups in which the rich club effects were detected across all subjects. As shown in Figure 1A, the values of the RC in the right hemisphere are significantly larger than those in the left hemisphere at the degree *k* = 5–6. The values of the RC in the NC group were higher than those in the BD group at degrees *k* = 4–5, as shown in Figure 1B. The normalized RCs increased with increasing nodal degrees *k* greater than 1 (Figures 1C,D), indicating that the rich-club organization existed in the hemispheric networks of both the NC and BD groups. Specially, we observed a specific *k* degree of 4 where normalized RC begin to be larger than 1. At degrees *k* = 5–8, significant group differences were observed in the normalized RCs as described in Figure 1D. However, we did not find any significant hemisphere differences in the normalized RCs, as presented in Figure 1C.

**FIGURE 2:**
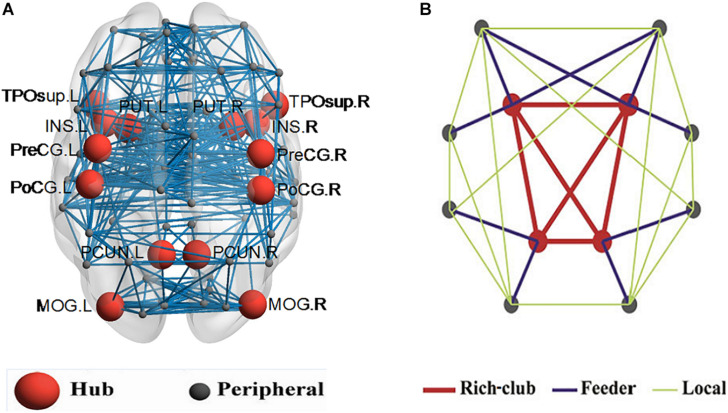
Hub nodes and the three types of connections in the rich club organization. **(A)** depicted hub nodes (red nodes) across all NC and BD groups. **(B)** depicted a simplified example of the three types of connections.

In this work, 16% of most consistently ranked nodes corresponding to the top 7 regions were defined as hub nodes across the two groups of subjects. In order of nodal degree, the seven regions were as follows: PUT, INS, PCUN, postcentral gyrus [PoCG], precentral gyrus [PreCG], temporal pole (superior) [TPOsup], middle occipital gyrus [MOG], illustrated in Figure 2A. The remaining 38 regions were classified as peripheral nodes. Based on the classification of the network nodes, the network edges were classified into three types of connections: rich-club connections linking two hub nodes, feeder connections linking hub and peripheral nodes, and local connections linking two peripheral nodes (Figure 2B).

### Group and Hemisphere Effects on Rich Club Organization

#### Three Classifications of Connections

**FIGURE 3:**
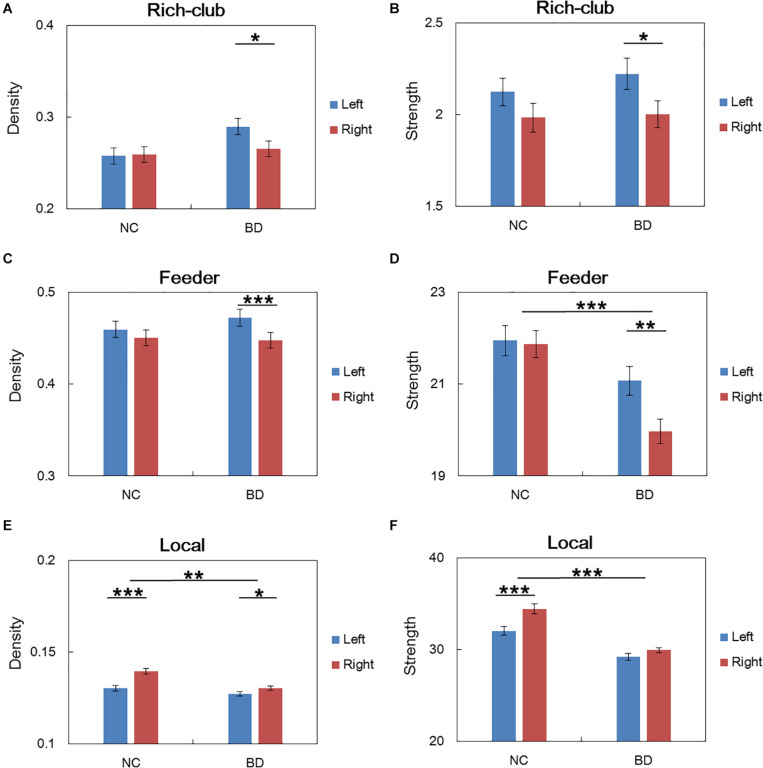
*Post hoc* analysis of the rich club organization. **(A,C,E)** depicted both significant hemisphere and group differences in the density of rich-club, feeder and local connections, respectively. **(B,D,F)** depicted both significant hemisphere and group differences in the strength of rich-club, feeder and local connections, respectively. NC: normal control. BD: bipolar disorder. *p < 0.05; **p < 0.01; ***p < 0.001.

For connections in rich club organization, significant group and hemisphere effects on the connectivity density and strength are depicted in Table 2 and Figure 3. As shown in Table 2, we found no significant group and hemisphere difference in the rich-club connections. Further analysis (Figure 3) showed smaller values for the connectivity measures in the BD patients than in the NCs, indicating disrupted feeder and local connections in BD. Table 2 shows that feeder and local connections exhibited significant hemisphere-by-group interactions. The *post hoc* analysis revealed that the hemisphere-by-group interaction resulted from different patterns of hemispheric asymmetry in the feeder and local connections in the two groups of subjects. Figures 3C,D shows that BD patients exhibited a significant left hemisphere advantage in the two connectivity measures of feeder connections. However, the NC group exhibited significant rightward asymmetry in both the connectivity density and strength of local connections, as shown in Figures 3E,F.

**TABLE 2:** Differences in the connectivity measures of the rich club organization.

Connectivity measure	Rich club organization	Group difference *F*_1_,_99_ (*p*-value)	Hemisphere difference *F*_1_,_99_ (*p*-value)	Interaction *F*_1_,_99_ (*p*-value)
Density	Rich-club	3.499 (0.064)	0.016 (0.901)	2.173 (0.144)
	Feeder	0.456 (0.501)	8.268 (**0.005**)	4.328 (**0.040**)
	Local	11.651 (**0.001**)	8.931 (**0.004**)	11.192 (**0.001**)
Strength	Rich-club	0.268 (0.606)	0.218 (0.642)	0.044 (0.835)
	Feeder	17.758 (<**0.001**)	4.122 (**0.045**)	5.765 (**0.018**)
	Local	47.759 (**<0.001**)	5.138 (**0.026**)	9.660 (**0.002**)

*Significant differences (p < 0.05) were indicated by the bold text.*

The statistical analysis results of the asymmetry scores in the two connectivity measures of the rich club organization in the two groups are shown in Table 3. For the NCs, only the local connections exhibited a significant right hemisphere advantage (positive AS, *p* < 0.001) in the connectivity density and strength. For the BD patients, the rich-club connections and feeder connections showed significant a left hemisphere advantage in the connectivity density and strength. In addition, the feeder and local connections showed evident group differences in the asymmetry scores of the connectivity density and strength. This group-difference finding is in accordance with the significant group-by-hemisphere interaction observed in the connections of the rich club organization.

**TABLE 3:** Analysis of the asymmetry scores for the connectivity measures.

Connectivity measure	Rich club organization	NC group *t*_*54*_ (*p*-value)	BD Patients *t*_*48*_ (*p*-value)	BD versus NC *t*_1_,_102_ (*p*-value)
Density	Rich-club	0.068 (0.946)	−2.687 (**0.010**)	1.710 (0.090)
	Feeder	−1.382 (0.173)	−4.096 (**<0.001**)	2.114 (**0.037**)
	Local	8.363 (**<0.001**)	2.064 (**0.044**)	2.972 (**0.004**)
Strength	Rich-club	−1.849 (0.070)	−2.638 (**0.011**)	0.101 (0.752)
	Feeder	0.019 (0.985)	−3.283 (**0.002**)	5.782 (**0.018**)
	Local	6.837 (**<0.001**)	1.695 (0.097)	7.220 (**0.008**)

*Significant differences (p < 0.05) were indicated by the bold text.*

**FIGURE 4:**
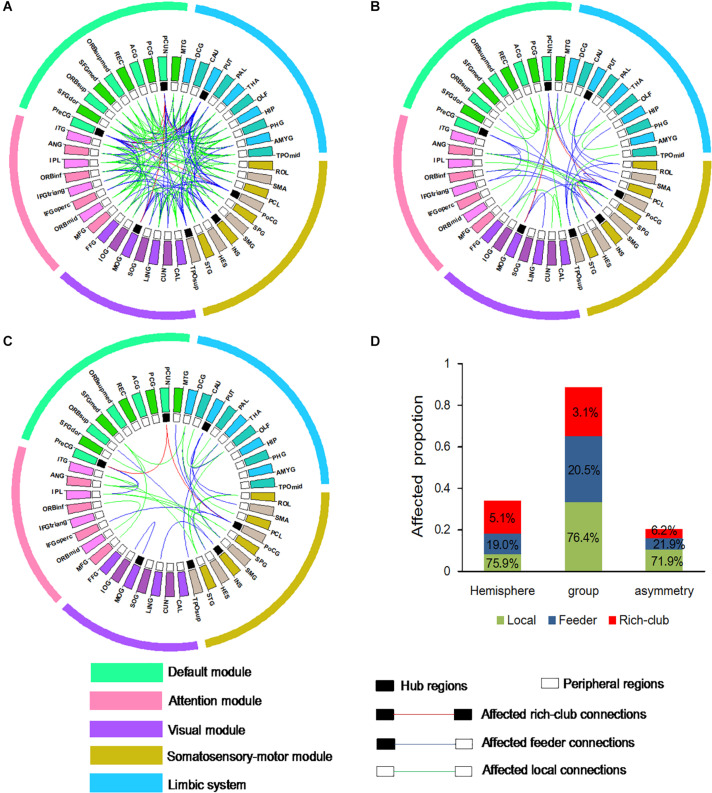
Aberrant WM connections **(A)** depicted aberrant WM connections with group differences between NC and BD. **(B)** Depicted aberrant WM connections with hemisphere differences between the left and right hemisphere. **(C)** Depicted aberrant WM connections with group difference in the hemispheric asymmetry of connection measures. Red edges indicate affected rich-club connections, blue edges indicate affected feeder connections, and green edges indicate affected local connections. The classification of hub nodes and peripheral nodes is depicted by the inner ring (black squares indicating hub nodes and white squares indicating peripheral nodes). **(D)** Proportion of significantly aberrant connections (100% × observed/total) illustrated by rich-club, feeder and local edges.

To examine whether connections is distributed among three classification of connections in the BD group compared to the NC group, and the left hemisphere compared to right hemisphere, an additional analysis was performed. Abnormal WM connectivity was observed in the BD group relative to the NC group, with 161 connections (5 rich-club, 33 feeder, and 123 local connections; Figure 4A). Significant differences between the left and right hemisphere were observed in 58 connections (3 rich-club, 11 feeder, and 44 local connections; Figure 4B). A significant group difference between NC and BD group was observed in the hemispheric asymmetry of WM connectivity, with 32 connections (2 rich-club, 7 feeder, and 23 local connections; Figure 4C). The proportion (100% × observed/total) of each classification of aberrant WM connections was illustrated in Figure 4D. These findings tend to suggest that the rich-club connections might be stable.

#### Nodal Degree of the Hub and Peripheral Regions

**FIGURE 5:**
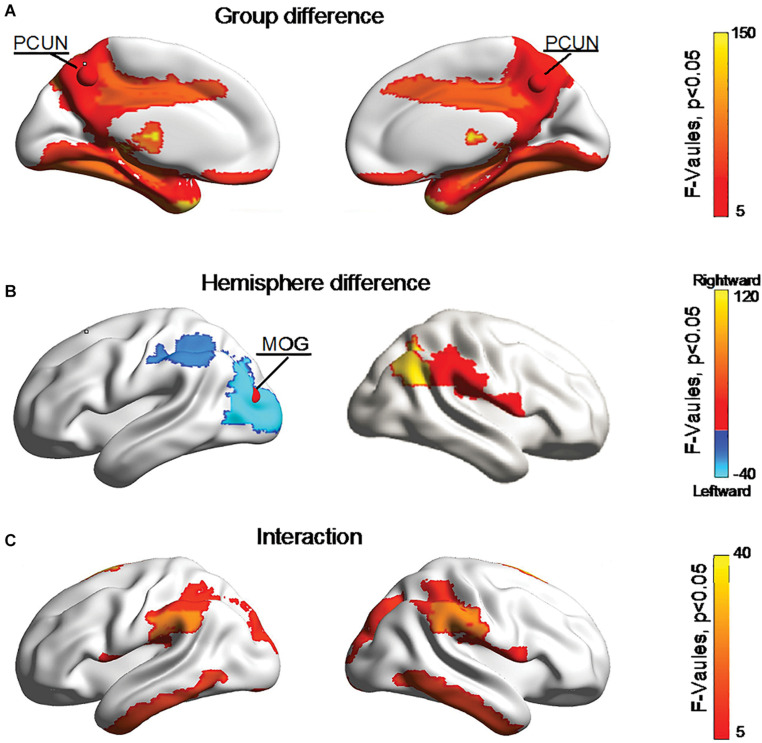
Bonferroni-corrected group and hemisphere differences in the nodal degrees of the hub regions (red nodes) and peripheral regions. **(A)** Depicted regions with significant group differences in nodal degree. **(B)** Depicted regions with significant hemisphere differences in nodal degree. **(C)** Depicted regions with significant group-by-hemisphere interactions in nodal degree.

**FIGURE 6:**
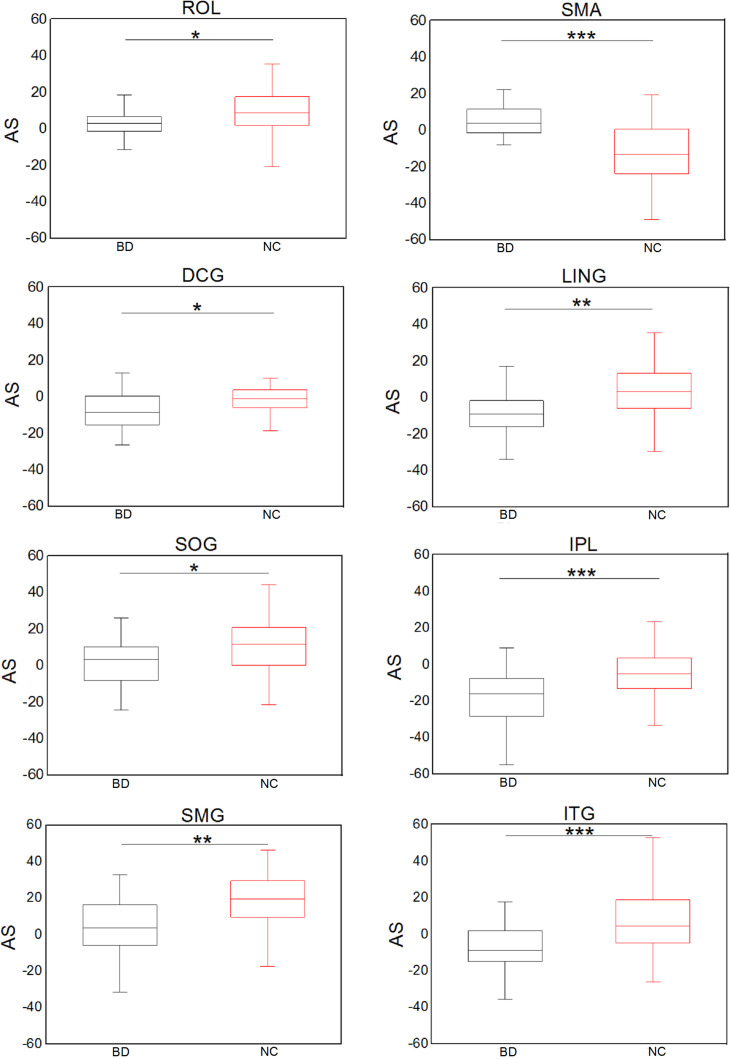
Significant group differences in the asymmetry score of the nodal degree. *p < 0.05; **p < 0.01; ***p < 0.001 (Bonferroni-corrected).

Figure 5 shows significant (Bonferroni-corrected, *p* < 0.05) group and hemisphere differences in the regional degree. Specifically, several regions including the PCUN hub region showed significant group differences in nodal degree, shown in Figure 5A. Figure 5B illustrated that eight regions including the MOG hub region exhibited prominent hemisphere differences in the nodal degree. Among these regions, the ACG, middle cingulate and paracingulate gyri [DCG], posterior cingulate gyrus [PCG], MOG and inferior parietal lobule [IPL] showed evident left-greater-than-right asymmetries in the nodal degree, whereas the ROL, the supramarginal gyrus [SMG] and the angular gyrus [ANG] exhibited more advantageous nodal degrees in the right hemisphere than the left hemisphere. Figure 5C showed significant group-by-hemisphere interactions in nodal degree of eight peripheral regions. The *post hoc* analysis indicated that these interaction effects resulted from significant group differences in the asymmetry score of regional degree between the two groups, as shown in Figure 6.

### Correlation With Symptoms of BD

**FIGURE 7:**
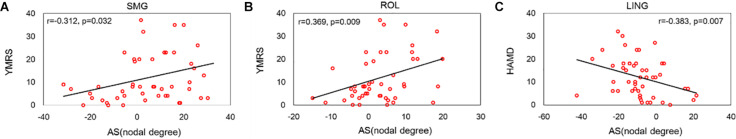
Significant correlations between the asymmetry score of the nodal degree and the symptoms of BD patients. **(A)** Depicted a positive relationship between the nodal degree of SMGregion and the score of YMRS symptom. **(B)** Depicted a positive relationship between the nodal degree of ROL region and the score of YMRS symptom. **(C)** Depicted a negative relationship between the nodal degree of LING region and the score of HAMD symptom.

This work investigated the relationship between the asymmetry score of abnormal nodal degrees of peripheral regions and the clinical symptoms of BD patients. Significant correlations are depicted in Figure 7. The ROL and SMG regions exhibited a positive relationship between the asymmetry score of the nodal degree and the YMRS score. Evident negative correlation was revealed between the asymmetry score of the LING regional degree and the HAMD score.

## Discussion

This work employed graph theory approaches to analyze abnormalities in the rich club organization of hemispheric WM brain networks in BD. Disrupted feeder and local connections were revealed in BD patients compared to NCs, which resulted in significantly leftward asymmetry in the feeder connections and decreased rightward asymmetry in the local connections in BD patients. Moreover, we found that the asymmetry scores of the abnormal nodal degrees were significantly correlated with the symptoms of BD.

### Rich Club Organization

The normalized RCs of the hemispheric WM networks increased and were greater than 1 over a range of degrees for the two groups of subjects, reflecting the existence of a rich club organization in the hemispheric WM networks (Yan et al., 2018; Wang et al., 2019). Moreover, the current work revealed a group of seven strongly interconnected hemispheric hub nodes comprising the PUT, INS, PCUN, PoCG, PreCG, TPOsup, and MOG regions for the two groups, largely consistent with previous studies on whole-brain networks (Collin et al., 2016; O’Donoghue et al., 2017; Wang Y. et al., 2018). Our findings suggested that the rich club organization existed in not only the whole-brain networks but also the hemispheric-brain networks (Wang et al., 2019).

### Disrupted Connections in the Rich Club Organization in BD

#### Disruption of the Feeder and Local Connections

This work defined the FA as the weight of the network edge. We showed that feeder connections exhibited significantly reduced the connectivity strength but not the density in BD patients, suggesting significantly decreased FA in BD patients compared with the NC group. Our finding is consistent with previous work (Collin et al., 2016; Collin et al., 2017), showing no disruptions in the rich-club connections. Moreover, previous DTI studies (Serene et al., 2010; Collin et al., 2016) on BD have reported FA reductions in the brain WM related to the NC group, suggesting our results are reasonable. The FA value expresses the coherence of the organization of fibers within a voxel and provides an index of the structural integrity of WM (Keller et al., 2007; Pierpaoli and Basser, 1996). Hence, disrupted feeder connections may provide evidence of decreased WM integrity in BD patients. Prior studies (Sussmann et al., 2009; Benedetti et al., 2011) have revealed disruptions of the WM integrity is a possible structural marker of BD.

In addition, BD patients showed significantly decreased connectivity density of local connections compared with the NC group, reflecting disrupted WM connections. As one of the graph indexes, the clustering coefficient measures the existing number of connections between the node and its nearest neighbors out of all possible connections (Bullmore and Sporns, 2009). Previous studies on structural networks have reported disrupted, lower clustering coefficients (Leow et al., 2013; O’Donoghue et al., 2017) in BD patients than NCs, suggesting disrupted WM connections. Moreover, this work revealed decreased connectivity strength of local connections in BD patients, reflecting reduced WM integrity. The network efficiency measures the integration ability of connections in brain networks (Yasser et al., 2011). Lower values of the network efficiency were observed in the WM networks of BD patients (Wang Y. et al., 2018), demonstrating reduced WM integrity. In summary, this study divided all the WM connections into three classifications and found significant disruption in both the feeder and local connections in BD patients. Our findings reflect that disruptions of feeder and local connections may result in abnormal WM connectivity in BD patients.

#### Disconnections in Hub and Peripheral Regions

We found that the hub region PCUN and several peripheral regions showed decreased nodal degrees in BD patients, suggesting that disconnections linked these regions. Functional (Strakowski et al., 2000) and structural (O’Donoghue et al., 2015) studies have revealed disconnections in the PCUN region. Moreover, previous WM studies (Cui et al., 2011; Martinot et al., 2014) have revealed a significant reduction in the FA in the PCUN region in BD patients. Notably, the PCUN region has an important role in the default mode network (DMN), which involved neurocognitive functions such as memory and attention (Delano-Wood et al., 2012). Combined with reduced nodal degree in the PCUN in BD, our findings may suggest that cognitive deficits may be more representative of BD (Maalouf et al., 2010).

This work revealed significant decrease in nodal degrees of several peripheral regions in BD patients. We observed that these abnormal regions (the ANG, middle temporal gyrus [MTG], inferior frontal gyrus (orbital part) [ORBinf], middle frontal gyrus (orbital part) [ORBmid], PCG, parahippocampal gyrus [PHG], rectus gyrus [REC], and middle temporal gyrus [TPOmid]) were predominantly located in the DMN. Consistently, studies (Forde et al., 2015; Wang et al., 2017) have reported aberrant connections in the DMN of BD patients. The DMN is believed to be involved in affective regulation (Kaiser et al., 2015). Aberrant connections in the DMN indicated impaired affective regulation function. Other abnormal regions, including inferior frontal gyrus (triangular) [IFGtriang], IPL, ITG and superior parietal gyrus [SPG] regions, are mainly located in control execution network (CEN). The CEN is responsible for high-level cognitive functions, such as attention and working memory (Menon, 2011). Our findings may reflect disrupted cognitive function in BD patients. Impairments in the function of the DMN and CEN in BD were revealed in previous structural (Wang Y. et al., 2018) and functional (Goya-Maldonado et al., 2016; Wang et al., 2016) studies. Hence, decreased nodal degrees of peripheral regions in the DMN and CEN may contribute to core deficits in cognitive and affective functioning in BD patients.

### Abnormal Asymmetry of Feeder and Local Connections in BD

This work revealed significant group differences in the hemispheric asymmetry of the feeder and local connections. We observed that feeder connections showed evident leftward asymmetry in the two connectivity measures in BD patients but disappeared in the NCs, as shown in Figure 3. Consistently, our previous work (Wang et al., 2019) on NCs did not find hemispheric asymmetry in connectivity measures of feeder connections. Significantly decreased rightward asymmetry in the two connectivity measures of local connections was found in BD patients compared with NC, suggesting evident disruption of WM connections of the right hemisphere. The predominantly right hemispheric disconnections in BD patients were consistent with the abnormal right lateralization of WM in BD patients (Vederine et al., 2011; Ho et al., 2017). It has been suggested that the right hemisphere is preferentially involved in emotions (Schwartz et al., 1975; Wheeler et al., 1993), visuospatial abilities (Cullen et al., 2016) and that disturbances of the right hemisphere underlie emotion dysregulation and visuospatial processing deficits (Caligiuri et al., 2004).

In addition, we found that several peripheral regions, including the IPL, ROL, LING, SOG, SMG and ITG, showed evident group differences in the asymmetry score of the nodal degree. Both feeder and local connections are linked to peripheral regions. Hence, asymmetry differences in these peripheral regions might contribute to abnormal hemispheric asymmetry in the feeder and local connections. Specifically, we found that the IPL, ITG and DCG regions showed evident leftward asymmetry of the nodal degree in BD patients. Accordingly, prior research (Liu et al., 2012) has shown left asymmetry in the regional homogeneity in the IPL region in BD. Structural researches (Hallahan et al., 2011; Lisy et al., 2011) have revealed left asymmetry of gray matter in the ITG region of BD patients. Functional research (Wang et al.) has showed increased functional connectivity in the left cingulate cortex and left temporal gyrus. These studies may provide support for our above-mentioned findings of leftward asymmetry in regional degree. Moreover, this work revealed that BD patients showed decreased rightward asymmetry in the nodal degree of the ROL, SOG, and SMG, suggesting that these regional degrees in the right hemisphere were disrupted. Notably, it has been demonstrated that the right ROL and SMG regions are involved in emotional regulation (Silani et al., 2013). A reduction in the nodal degree of these two regions may reflect impaired emotional regulation in BD. It was proven that alteration of emotional regulation is one of significant symptoms in BD patients (Pavuluri et al., 2006), demonstrating that our findings are reasonable. The SOG is associated with visuospatial processing (Green, 2006; Bearden et al., 2015). The reduced rightward asymmetry of the nodal degree in these regions might be associated with deficits in emotional and visuospatial functions (Bearden et al., 2015).

### Clinical Correlation

We found that the asymmetry score of the ROL and SMG regional degree showed a positive relationship with the YMRS symptom score. Consistent with our findings, Gao et al. (2017) found that connectivity in the right ROL was positively associated with BD features. One research (Jie et al., 2015) reported that connections linking the ROL and SMG regions correlated with the BD feature. The LING region showed a negative relationship with the HAMD score of HAMD, suggesting that the number of LING-based connections decreases with increasing HAMD score. A previous research (Lv et al., 2016) revealed that the decreased connectivity strength linked to the right LING region showed a significant positive correlation with the scores on the HAMD. These studies show that our findings are reasonable and correct. Specially, these three regions are defined as peripheral regions in this work. Hence, our correlation results may provide support for our findings of no disruption in rich-club connections and abnormal hemispheric asymmetries as a marker of BD.

## Conclusion

This work examined changes in the rich club organization of the hemispheric WM networks in BD. We revealed no disruption in the rich-club connections but significantly disrupted feeder and local connections in BD patients. Moreover, these abnormal connections involving regions in DMN and CEN supported impaired attention, working memory and affective functioning in BD patients. In addition, aberrant asymmetry in the feeder and local connections was found in BD patients, which might be related to emotional regulation and visuospatial functions. The correlation results showed that the abnormal asymmetry of peripheral regional degree was related to clinical symptoms in BD patients. These findings highlight the potential for stable rich-club connections but not feeder and local connections in the rich club organization of hemispheric WM networks in BD patients. This work provides another perspective for understanding the pathological mechanisms of BD.

## Data Availability Statement

Publicly available datasets were analyzed in this study. This data can be found here: https://www.openfmri.org/.

## Ethics Statement

The studies involving human participants were reviewed and approved by Institutional Review Board of the University of California, Los Angeles (UCLA). The patients/participants provided their written informed consent to participate in this study. Written informed consent was obtained from the individual(s) for the publication of any potentially identifiable images or data included in this article.

## Author Contributions

DL completed the entire study of the experiment and writing. WL, JW, YM, and NZ revised the manuscript. TY, XC, ZZ, and JX provided advice and guidance. BW provided the research ideas. ZZ completed the interpretation data, revision of the manuscript and provided critical suggestions for our manuscript proof. All authors contributed to the article and approved the submitted version.

## Conflict of Interest

The authors declare that the research was conducted in the absence of any commercial or financial relationships that could be construed as a potential conflict of interest.

## Footnotes

1 https://www.openfmri.org/

2 http://www.nitrc.org/projects/gretna/

3 http://www.nitrc.org/projects/bnv

4 http://www.spss.com/
