# Supplementary material for: Disrupted Rich Club Organization of Hemispheric White Matter Networks in Bipolar Disorder
Source: Front Neuroinform. 2020 Aug 26;14:39. doi: 10.3389/fninf.2020.00039 (PMC7479125; doi:10.3389/fninf.2020.00039)
Supplement: Supplementary file 1 [file Table_1.DOC]

**We added the standard deviation of RC for per hemisphere and group. In order to better represent the hemispheric or group differences, we add standard error based on bar chart. The bar chart is shown in the supplementary materials.**

**
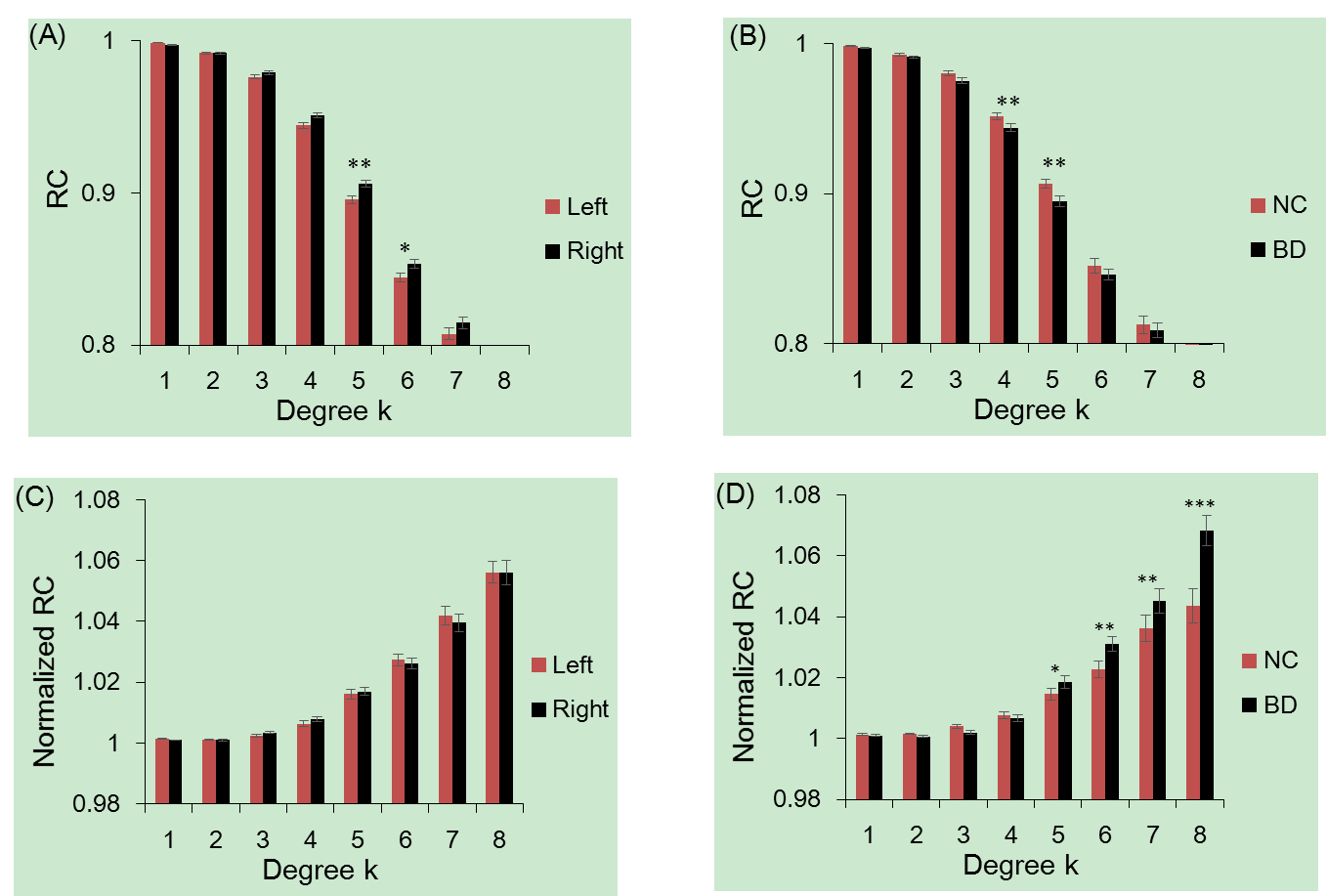
**

**Figure 1. Hemisphere and group differences in both the RCs and normalized RCs. Figure 1(A) and 1(B) depicted significant hemisphere and group differences in RC, respectively. Figure 1(C) and 1(D) depicted significant hemisphere and group differences in normalized RC, respectively. *: p <0.05; **: p<0.01; ***: p<0.001**
